# Updates in the 2025 AHA/ACC Hypertension Guideline

**DOI:** 10.1007/s11906-026-01372-9

**Published:** 2026-03-17

**Authors:** Carly Brown, Donald Clark, Daniel W. Jones

**Affiliations:** 1 IMPACT Center for Pharmacy Transformation, University of Mississippi School of Pharmacy, Jackson, MS USA; 2 Department of Medicine, University of Mississippi Medical Center, 2500 North State Street, Jackson, MS 39216 USA

**Keywords:** Hypertension management, AHA/ACC 2025 guideline, Cardiovascular risk assessment, Resistant hypertension, Patient-centered care

## Abstract

**Purpose of Review:**

Hypertension remains a leading cause of cardiovascular morbidity and mortality in the United States. The 2025 American Heart Association (AHA)/American College of Cardiology (ACC) Guideline for the Management of High Blood Pressure in Adults provides the first major update since 2017. It reveals new evidence in the diagnosis, risk assessment, and treatment of hypertension. This review summarizes key updates, contrasts them with prior recommendations, and discusses implications for clinical practice.

**Recent Findings:**

The 2025 AHA/ACC Hypertension Guideline introduces several major updates that reshape risk assessment, diagnosis, and treatment strategy. Adoption of the PREVENT risk calculator replaces the pooled cohort equations, improving cardiovascular risk estimation by incorporating renal function, statin use, and social drivers of health. Screening for primary aldosteronism is expanded to all patients with stage 2 or resistant hypertension, addressing underdiagnosis of this secondary cause. Renal denervation is introduced as an adjunctive option for select patients with resistant hypertension. The guideline also provides expanded recommendations for special populations, including those with chronic kidney disease, diabetes, pregnancy, and neurologic disorders. Enhanced emphasis on home blood pressure monitoring, lifestyle modification, and team-based care highlights the growing importance of patient engagement and coordinated care.

**Summary:**

The 2025 AHA/ACC Hypertension Guideline marks a pivotal shift toward risk-based, patient-centered hypertension management. It reinforces the need for tailored strategies that bridge clinical practice and population health. Effective implementation will depend on interdisciplinary collaboration and equitable access to preventative and therapeutic resources.

## Introduction

Hypertension remains a leading risk factor for cardiovascular disease (CVD), stroke, and end stage renal disease, affecting an estimated 120 million adults in the United States [1, 2]. The American College of Cardiology (ACC) and the American Heart Association (AHA) have played a central role in translating scientific evidence into clinical practice guidelines for decades [3]. These guidelines standardize diagnosis, risk assessment, and treatment, ensuring consistent and evidence-based care across diverse populations. The 2017 AHA/ACC Hypertension Guideline marked a paradigm shift by lowering the threshold for hypertension diagnosis to ≥ 130/80 mm Hg, emphasizing early intervention and redefining control targets across populations [4].

Yet the need for updated guidance has grown more urgent. Nearly half of U.S. adults have hypertension, with prevalence rising over the past decade [5, 6]. Despite this burden, fewer than 60% are aware of their condition, nearly half receive treatment, and only one in five achieve blood pressure (BP) control [5]. This persistent gap highlights a major opportunity. Even modest improvements in prevention and control could yield substantial reductions in morbidity, mortality, and healthcare costs [7].

The newly released 2025 AHA/ACC Guideline builds on these foundations while addressing the limitations revealed through real-world implementation. It incorporates new evidence on secondary hypertension, refined risk estimation tools, comorbid condition management, and novel therapies such as renal denervation. These updates reflect the growing body of evidence since 2017 and address gaps in practice that emerged as clinicians applied prior recommendations.

The economic burden of hypertension highlights the stakes of inadequate control. CVD accounted for over $400 billion in direct healthcare expenditures and lost productivity in 2020–2021, with hypertension representing a major contributor to these costs [8]. Reducing this burden will require more effective strategies to improve control. Priorities include expanding access to preventive care, greater use of home and ambulatory BP monitoring, and careful evaluation of lifestyle, pharmacologic, and device-based approaches [9].

This review summarizes significant updates in the 2025 AHA/ACC Hypertension Guideline, contrasting them with the 2017 guideline, and explores their implications for clinical practice and public health (Table 1). Special attention is given to how these changes collectively reflect a more risk-adapted, patient-centered model of hypertension care.

**Table 1:** Comparison of major updates

Major Update	2017 Guideline Approach	2025 Guideline Approach	Key Takeaway
Structural and Terminology Framework	Separate sections for race/ethnicity, sex, and age; use of “hypertensive urgency”	Integrated discussion of demographic factors across evidence base; adoption of “severe hypertension”	Shift toward integrated cardiovascular risk assessment and updated terminology
Secondary Hypertension Evaluation	Targeted screening for select high-risk groups; hypokalemia emphasized	Expanded screening for primary aldosteronism regardless of potassium levels; broader eligibility criteria for testing	Emphasis on underdiagnosis and earlier detection of secondary hypertension, particularly primary aldosteronism
Lifestyle Modification	Foundational but variably specified	Evidence-based lifestyle strategies including salt substitutes	More directive nonpharmacologic management
Diagnostic Testing and Monitoring	Office BP with supportive use of HBPM/ABPM for monitoring	HBPM and ABPM central to diagnosis and management; structured monitoring guidance	Greater emphasis on diagnostic accuracy and longitudinal engagement
Risk Estimation and Treatment Thresholds	Pooled Cohort Equations; 10-year ASCVD risk	PREVENT calculator; 10- and 30-year total CVD risk	More refined risk stratification guiding treatment intensity
Resistant Hypertension Therapies	Focus on medication optimization	Renal denervation introduced as Class IIb adjunctive option	Inclusion of device-based therapies with shared decision-making
Special Populations	Limited population-specific guidance	Expanded guidance for CKD, diabetes, neurologic disease, cognition, and pregnancy	Greater personalization and safety-focused targets
Complications of Management	Cautious BP lowering in select scenarios	Emphasis on avoiding overtreatment while maintaining CV benefit	Balanced approach to intensive BP lowering

*ASCVD* indicates atherosclerotic cardiovascular disease, *ABPM* ambulatory blood pressure monitoring, *BP* blood pressure, *CKD* chronic kidney disease, *CV* cardiovascular, *CVD* cardiovascular disease, *HBPM* home blood pressure monitoring, *PREVENT* Predicting Risk of CVD EVENTs

## Overview of the 2025 AHA/ACC Guidelines

The 2025 AHA/ACC Guideline for the Management of High Blood Pressure in Adults serves as a comprehensive resource for clinical and public health professionals, integrating emerging evidence and implementation strategies to improve hypertension prevention and control. Replacing the 2017 guideline, the new document expands its scope, refines diagnostic criteria, and adopts advanced risk estimation tools that align treatment decisions with overall cardiovascular risk. The guideline writing committee was comprised of 28 clinicians. There are 11 collaborating organizations.

In developing the guideline, the writing committee conducted a comprehensive review of scientific evidence and prior recommendations, identifying the most effective approaches to BP estimation, risk prediction, and management in adults with and without established CVD. A central advance in the 2025 guideline is the adoption of the PREVENT (Predicting Risk of CVD Events) model for risk estimation. PREVENT estimates total cardiovascular risk in adults aged 30–79 years, incorporating statin use, kidney function, and social drivers of health such as the social deprivation index [3]. This refinement improves predictive accuracy compared with the pooled cohort equations (PCEs) and supports more individualized thresholds and treatment goals.

While many core principles from the 2017 guideline remain, the 2025 update strengthens emphasis in several areas critical to both clinical practice and population health. The overarching treatment goal of < 130/80 mm Hg is reaffirmed with “encouragement to achieve < 120/80 mm Hg” for most adults [3]. Exceptions include those in institutional care, with limited life expectancy, or who are pregnant.

Lifestyle interventions including weight management, sodium restriction, DASH-style eating, physical activity, stress management, and alcohol reduction remain first-line therapy and are further emphasized as foundational strategies. Beyond lifestyle counseling, the guideline reinforces the need for system-level interventions to include partnerships between clinicians, community organizations, and health systems to expand access to screening and preventive resources.

Collaboration and team-based care also receive expanded attention. The 2025 guideline highlights multidisciplinary models involving physicians, nurses, pharmacists, dietitians, and community health workers as critical for achieving sustained BP control. These strategies acknowledge that hypertension management extends beyond individual clinical encounters and depends on coordinated systems of care.

Importantly, the guideline points out that benefits for intensive lowering of BP not only include lower risk for heart disease, stroke, and kidney disease, but also dementia risk is reduced [10].

Collectively, the updates represent both continuity and advancement. The 2025 recommendations preserve the evidence-based precision of the 2017 guideline while expanding the framework to encompass risk stratification, social determinants of health, and multidisciplinary collaboration. Together, these refinements embody a more integrated, patient-centered model of hypertension management that bridges clinical precision with population impact.

## Summary of Major Updates

### Structural and Terminology Changes

In contrast to the 2017 version, the 2025 guideline introduces structural and terminology updates that reflect a broader, more integrated approach. One notable modification is the removal of dedicated sections on race/ethnicity, sex-specific considerations, and age-related issues, which were prominent. Rather than isolating these categories, the new framework embeds them throughout its broader evidence discussions, underscoring that such factors remain critical but should be interpreted in the context of overall cardiovascular risk.

Another key change is the replacement of the term hypertensive urgency with severe hypertension [3]. This modification reflects a more precise description of patients who present with markedly elevated BP (> 180/120 mmHg) without acute target-organ damage [3]. It aligns terminology with contemporary clinical practice and reduces ambiguity in emergency settings.

### Secondary Hypertension and Evaluation

Building on these structural refinements, the 2025 guideline expands its focus on secondary hypertension, particularly primary aldosteronism (PA) by emphasizing its growing recognition and underdiagnosis. PA accounts for approximately 5%–10% of all hypertension and up to 20% of resistant hypertension cases, yet screening rates among eligible patients remain below 2% [11, 12]. Accordingly, the guideline now recommends screening all patients with stage 2 or resistant hypertension, regardless of serum potassium levels, recognizing that hypokalemia is absent in most cases [13, 14]. In addition, expanded testing criteria also includes patients with hypertension accompanied by hypokalemia (spontaneous or diuretic-induced), muscle cramps or weakness, an incidentally discovered adrenal mass, obstructive sleep apnea, or a family history of early-onset hypertension or stroke [11, 13–16]. The heightened emphasis on screening for PA moves this guideline closer to Endocrine Society recommendations which support testing in all patients with any stage of hypertension [17]. 

Initial and confirmatory testing protocols remain largely consistent with the 2017 guideline with the addition of the urine albumin to creatinine ratio. Screening for primary aldosteronism involves measurement of plasma aldosterone concentration (PAC), plasma renin activity (PRA), and calculation of the aldosterone-to-renin ratio (ARR) [11, 18]. An ARR ≥ 30 (PAC in ng/dL, PRA in ng/mL/h) with PAC ≥ 10 ng/dL and suppressed renin activity (< 1 ng/mL/h) suggests PA and warrants confirmatory testing [11, 16, 18, 19]. Serum potassium should be normalized and salt intake unrestricted before screening [16]. Most antihypertensive medications can be continued during testing, including ACE inhibitors, ARBs, and calcium channel blockers [16]. Beta-blockers and central alpha-agonists can suppress renin and may cause false-positive results, but results can still be interpreted in most cases [16]. Mineralocorticoid receptor antagonists (MRAs) such as spironolactone and eplerenone should be withheld for at least four weeks prior to testing [16]. If results are ambiguous, interfering agents may be temporarily substituted with noninterfering medications such as nondihydropyridine calcium channel blockers, vasodilators, or peripheral alpha-blockers for a minimum of two to four weeks before repeating the test [20].

### Lifestyle Modification and Monitoring

Complementing its diagnostic refinements, the 2025 guideline reinforces and strengthens the central role of lifestyle interventions in BP control. New evidence supports the use of potassium-based salt substitutes for select patients, with caution in those with severe chronic kidney disease (CKD) or reduced potassium excretion, while continued attention is given to sodium restriction, physical activity, reduced alcohol intake, and stress reduction [21–25].

Out-of-office BP measurement remains a cornerstone of diagnosis and management. Both home blood pressure monitoring (HBPM) and ambulatory blood pressure monitoring (ABPM) are recommended for confirming hypertension diagnosis [26, 27]. In addition, HBPM is recommended for ongoing management and medication titration [28, 29]. The guideline emphasizes identifying white-coat and masked hypertension, with updated guidance on monitoring frequency and technique to enhance diagnostic accuracy and patient engagement. Both the 2017 and 2025 guidelines stress that excluding the white-coat effect is critical before intensifying therapy, particularly in apparent resistant hypertension. Detecting masked hypertension is essential for avoiding undertreatment. Given mixed evidence and lack of external validations on cuffless BP devices, they are not recommended for diagnosing or treating elevated BP [30–32].

### BP Treatment Thresholds and Risk Estimation

Perhaps the most conceptually significant update is the adoption of the PREVENT risk calculator in place of the PCEs for cardiovascular risk estimation [33]. While the PCEs estimated the 10-year risk of atherosclerotic cardiovascular disease in adults aged 40–79 years not on statin therapy, PREVENT estimates 10 year and 30 year total CVD risk, including myocardial infarction, stroke, and heart failure, in adults aged 30–79 years. PREVENT incorporates additional predictors such as kidney function, statin use, and place-based social drivers of health. These refinements enhance risk stratification and better align treatment intensity with individual cardiovascular profiles.

For adults with previous CVD, diabetes, CKD, or a ≥ 7.5% 10-year CVD risk, pharmacologic treatment is now recommended when BP is ≥ 130/80 mm Hg [34–39]. For those with < 7.5% risk, medication initiation is reserved for patients who remain ≥ 130/80 mm Hg after 3–6 months of lifestyle intervention [36, 38, 39]. This stepwise, data-driven approach exemplifies the guideline’s overarching theme—aligning treatment intensity with individual risk.

For adults with stage 2 hypertension (≥ 140/90 mm Hg), the guideline recommends initiating treatment with two first-line antihypertensive agents of different classes, preferably in a single-pill, fixed-dose combination. This approach improves adherence and reduces the time needed to achieve BP control compared with prescribing separate medications [40–42].

### Resistant Hypertension and Renal Denervation

Expanding on pharmacologic strategies, the 2025 guideline introduces renal denervation (RDN) as a potential adjunctive therapy for patients with resistant hypertension. RDN is now recognized as a potential option for adults whose BP remains uncontrolled (office systolic BP 140–180 mm Hg and diastolic BP ≥ 90 mm Hg) despite optimized pharmacologic therapy or who are unable to tolerate multiple agents [43–45].

Evidence from sham-controlled trials demonstrates that RDN lowers 24-hour systolic BP by approximately 4–6 mm Hg in patients not on medications and by 3–5 mm Hg in those already treated with two to five antihypertensive drugs, although efficacy has varied across studies [44–51]. Most clinical trials enrolled participants with both systolic and diastolic hypertension, an estimated glomerular filtration rate ≥ 40 mL/min/1.73 m², and suitable renal artery anatomy (3–8 mm diameter), excluding those with renal artery stenosis, fibromuscular dysplasia, renal artery aneurysm, or prior stenting [44–48, 52]. Approximately 60%–70% of treated patients experienced a meaningful reduction of ≥ 5 mm Hg in ambulatory systolic BP [43, 53].

In the updated guideline, RDN receives a Class IIb recommendation (“may be considered”) for adults with resistant hypertension despite optimal medical therapy or those intolerant to multiple agents. The procedure should be performed by an experienced interventionalist after specialist evaluation to confirm eligibility and exclude contraindications. Post-procedure, noninvasive imaging is recommended to monitor for renal artery stenosis, which occurs in approximately 0.2% of patients per year with the highest risk in the first 6 months [54]. Shared decision-making is essential, as long-term cardiovascular outcome data remain limited. RDN should not be viewed as curative or as a replacement for antihypertensive medications.

### Special Populations

Continuing the guideline’s focus on personalization, the 2025 document delivers some of its most detailed revisions for special populations. For patients with diabetes and CKD, stronger recommendations are made for renin–angiotensin system inhibitors. ACE inhibitors or ARBs are preferred in those with albuminuria ≥ 30 mg/g or eGFR < 60 mL/min/1.73 m² to reduce CVD risk and slow kidney disease progression [55–57]. Although a treatment goal of systolic BP < 130 mmHg is recommended, there is additional encouragement to reach a systolic BP of < 120 mmHg to further reduce CVD morbidity and mortality [58–62].

Neurologic considerations include updated BP targets across several conditions. For acute spontaneous intracerebral hemorrhage (ICH), the 2025 guideline now recommends lowering systolic BP to 130 to < 140 mm Hg for at least 7 days in patients presenting with systolic BP 150 to 220 mm Hg, with careful titration to avoid excessive variability; therapy should be withheld if systolic BP falls below 130 mm Hg [63–65]. This contrasts with 2017, when recommendations focused only on lowering systolic BP > 220 mm Hg with intravenous infusion and cautioned against routine early lowering in the 150 to 220 mm Hg range. In acute ischemic stroke, the 2025 guideline specifies that reducing systolic BP < 140 mm Hg after successful endovascular reperfusion is harmful, updating the 2017 statement that aggressive lowering in spontaneous ICH within 6 h was not beneficial [66–68]. For cognition, the recommendation has been strengthened. The 2025 guideline endorses a firm systolic BP goal of < 130 mm Hg to reduce the risk of mild cognitive impairment and dementia, while the 2017 guideline suggested BP lowering was reasonable to prevent decline [10, 69–72].

In pregnancy, antihypertensive therapy should be initiated promptly for systolic BP ≥ 160 mm Hg or diastolic BP ≥ 110 mm Hg when confirmed on repeat measurements within 15 min and receive medications within 30 to 60 min to prevent complications [73–77]. Chronic hypertension should be managed to a goal of < 140/90 mm Hg to prevent worsening of maternal and perinatal morbidity and mortality [78–80]. Low-dose aspirin is advised beginning at 10–12 weeks of pregnancy to prevent preeclampsia in patients with hypertension or at risk of developing pre-eclampsia [81]. The list of contraindicated agents has been expanded to include atenolol, ACE inhibitors, ARBs, direct renin inhibitors, nitroprusside, and MRAs [82–86]. The updated timing for medication initiation and expanded contraindicated medication lists further illustrate the shift toward safety-tailored, patient-centered management.

### Complications of Management

Finally, the 2025 guideline addresses several management complications, reinforcing the importance of careful titration rather than reflexive intervention. The guideline recommends improved BP control in adults with hypertension to lower the risk of orthostatic hypotension (OH) [87–90]. In patients undergoing intensive BP-lowering therapy who develop asymptomatic OH, continuation of treatment to achieve a target systolic BP < 130 mm Hg is considered reasonable given the associated cardiovascular and mortality benefits [89, 91]. Furthermore, when initiating or intensifying antihypertensive therapy with a goal of systolic BP < 130 mm Hg, assessment for symptomatic OH is advised to help identify underlying chronic conditions [87–90].

In addition, a new recommendation addresses the management of severe hypertension in nonpregnant, nonstroke patients hospitalized for noncardiac conditions. In adults presenting with systolic BP > 180 mm Hg or diastolic BP > 120 mm Hg without evidence of acute target organ damage, the guideline advises against the intermittent use of intravenous or oral antihypertensive medications solely for the purpose of acute BP reduction [91–94]. Patients with symptoms or signs of acute organ damage should be hospitalized and managed with intravenous therapy.

## Clinical Implications

The 2025 AHA/ACC hypertension guideline carries important implications for daily clinical practice. The adoption of the PREVENT risk calculator enables clinicians to more accurately estimate total cardiovascular risk and tailor treatment thresholds to individual patient profiles, though this will require integration into clinical workflows and electronic health records. Embedding PREVENT into clinical decision support can streamline application at the point of care while also ensuring that social risk factors are consistently incorporated into treatment decisions.

Expanded recommendations for secondary hypertension, particularly universal screening for primary aldosteronism in stage 2 and resistant hypertension, emphasize the need for systematic evaluation and collaboration with specialty services. Stricter BP targets for patients with diabetes, CKD, and neurologic complications, as well as updated guidance for pregnancy, enables clinicians to align treatment thresholds with patient-specific risk and comorbidities, requiring close interdisciplinary coordination.

The 2025 guideline places greater emphasis on extending hypertension management beyond the clinic. While prior recommendations encouraged team-based care, the new framework integrates this model into nearly every aspect of implementation—from medication access to lifestyle counseling and community outreach. For clinicians, this means that improving control rates will depend less on individual provider encounters and more on coordinated systems of care. Telehealth is recommended as an important part of team based care.

Patient monitoring is another cornerstone. Routine use of standardized home BP monitoring, supported by frequent contact with care teams, can accelerate medication titration, reinforce adherence, and reduce therapeutic inertia. Therapy optimization remains critical. For adults with stage 2 hypertension, early initiation of dual therapy with single-pill combinations can shorten the time to control, while early outpatient management of severe hypertension avoids unnecessary hospitalizations and resource use.

Taken together, these strategies illustrate that translating the 2025 recommendations into scalable workflows will require not only new tools and thresholds but also redesigned systems of care. By pairing risk-based decision making with multidisciplinary, community-anchored, and equity-focused approaches, clinicians and health systems can improve BP control and reduce the disproportionate burden of CVD across diverse populations.

## Gaps and Future Directions

Despite the expanded scope of the 2025 AHA/ACC hypertension guideline, several important knowledge gaps remain. Awareness and optimal management of high BP continue to be suboptimal. Key uncertainties include the management of isolated diastolic hypertension, which is more common in younger populations, as evidence remains insufficient to define clear treatment thresholds and targets. Similarly, the ideal BP goals for various subpopulations, including those defined by age, comorbidities, and social determinants of health, require further clarification. Additional gaps include the role of sleep apnea treatment in lowering BP and the long-term cardiovascular risk.

Gaps also persist around BP measurement. Although home and ambulatory monitoring are emphasized, disparities in access to ABPM limit widespread implementation. The accuracy and clinical utility of wearable and cuffless devices remain unproven, and further studies are needed to compare measurement methods, including attended versus unattended automated office BP readings.

Further research should prioritize pragmatic trials to evaluate screening strategies, treatment thresholds, and implementation approaches in real-world settings beyond academic centers. For younger adults with diastolic hypertension, studies incorporating surrogate endpoints such as left ventricular hypertrophy and other measures of target-organ damage may offer feasible alternatives to large outcome trials. Investigations should also explore the integration of HBPM with health technology interventions to address nonadherence, as well as strategies to reduce disparities related to race, ethnicity, insurance status, and social determinants of health. Further research is needed to define the roles of genetic, epigenetic, environmental, and lifestyle factors in the development and progression of hypertension, particularly the contribution of obesity and the potential impact of emerging weight management therapies such as GLP-1 agonists. Addressing these gaps will be critical to refining future guideline recommendations, improving patient-centered hypertension care, and reducing the burden of CVD.

## Conclusion

The 2025 AHA/ACC Hypertension represents a meaningful evolution in the prevention and management of hypertension. It refines terminology, strengthens out-of-office monitoring, expands recommendations for secondary and resistant hypertension, and incorporates the PREVENT risk calculator to enhance risk estimation and guide treatment thresholds. By integrating these elements, the guideline shifts from a threshold-based framework to a more flexible, risk-adapted approach that aligns treatment intensity with each patient’s cardiovascular profile. Key updates for special populations further reinforce the need for context-specific strategies rather than uniform targets.

Successful implementation will depend on translating these recommendations into daily practice through interdisciplinary teamwork, patient engagement, and equitable access to diagnostic and therapeutic resources. Continued research and real-world application will be essential to sustain this progress and ensure future guidelines build on the foundation of precision and practical implementation set forth in 2025.

## Data Availability

No datasets were generated or analysed during the current study.
